# Temporal Dynamics of Religion as a Determinant of HIV Infection in East Zimbabwe: A Serial Cross-Sectional Analysis

**DOI:** 10.1371/journal.pone.0086060

**Published:** 2014-01-20

**Authors:** Rumbidzai Manzou, Christina Schumacher, Simon Gregson

**Affiliations:** 1 Manicaland HIV/STI Prevention Project. Biomedical Research and Training Institute, Harare, Zimbabwe; 2 Department of Infectious Disease Epidemiology, Imperial College London, London, United Kingdom; 3 Department of Pediatrics, Johns Hopkins University School of Medicine, Baltimore, Maryland, United States; Indiana University and Moi University, United States of America

## Abstract

**Background:**

Religion is an important underlying determinant of HIV spread in sub-Saharan Africa. However, little is known about how religion influences changes in HIV prevalence and associated sexual behaviours over time.

**Objectives:**

To compare changes in HIV prevalence between major religious groups in eastern Zimbabwe during a period of substantial HIV risk reduction (1998–2005) and to investigate whether variations observed can be explained by differences in behaviour change.

**Methods:**

We analysed serial cross-sectional data from two rounds of a longitudinal population survey in eastern Zimbabwe. Univariate and multivariate logistic regression models were developed to compare differences in sexual behaviour and HIV prevalence between religious groups and to investigate changes over time controlling for potential confounders.

**Results:**

Christian churches were the most popular religious grouping. Over time, Spiritualist churches increased in popularity and, for men, Traditional religion and no religion became less and more common, respectively. At baseline (1998–2000), HIV prevalence was higher in Traditionalists and in those with no religion than in people in Christian churches (men 26.7% and 23.8% vs. 17.5%, women: 35.4% and 37.5% vs. 24.1%). These effects were explained by differences in socio-demographic characteristics (for Traditional and men with no religion) or sexual behaviour (women with no religion). Spiritualist men (but not women) had lower HIV prevalence than Christians, after adjusting for socio-demographic characteristics (14.4% vs. 17.5%, aOR = 0.8), due to safer behaviour. HIV prevalence had fallen in all religious groups at follow-up (2003–2005). Odds of infection in Christians reduced relative to those in other religious groups for both sexes, effects that were mediated largely by greater reductions in sexual-risk behaviour and, possibly, for women, by patterns of conversion between churches.

**Conclusion:**

Variation in behavioural responses to HIV between the major church groupings has contributed to a change in the religious pattern of infection in eastern Zimbabwe.

## Introduction

Religion can help shape the behavioural norms within a society and the behaviours and practices of individuals.[1]–[3] Differences in religious composition, therefore, may contribute to the differences in the spread of HIV infection that have been observed between and within countries in sub-Saharan Africa.[3]–[7] In particular, religious beliefs and teachings may act as social enablers that facilitate the spread and adoption of messages promoted by national AIDS control programmes or, in some cases, may act as barriers to the adoption of these messages. [8], [9].

In Zimbabwe, HIV prevalence has fallen substantially from a peak of 27% in 1997 to around 14% currently. [10] This decline has been shown to have resulted from reductions in sexual risk behaviour (mainly multiple sexual partners) occurring most rapidly between 1998 and 2005. [11] These reductions in risk behaviour have, in turn, been attributed to increased awareness of AIDS deaths backed up by community-based HIV prevention programmes using school, workplace, church, peer education and other inter-personal communication activities. [12] For example, Gregson et al. showed that women who attended their local community group meetings (including church meetings) were more likely to have adopted lower-risk behaviours. [13].

Numerous different churches exist within Zimbabwe, which vary in their beliefs, teachings and practices on sexual, and health-seeking behaviour. [14], [15] It is important to establish whether there have been differences in the extent to which the HIV epidemic has affected members of these churches or in the extent to which different churches have been able to support effective responses to the epidemic, which have helped to reduce infection rates amongst their members over time. Data on any such differences would be useful for national programmes in working with different churches in a more focused way to control the HIV epidemic and its effects. However, to date, few published studies have compared the associations between HIV and specific religious groups in Zimbabwe.

In this study, we use data from an on-going longitudinal survey in the Manicaland region of Zimbabwe to determine: (1) whether differences existed in HIV prevalence between major religious groupings at the start of the HIV decline in Zimbabwe, (2) whether these differences were mediated by differences in past sexual risk behaviour, and (3) whether differences in sexual behaviour change contributed to variation in reductions in HIV prevalence between religious groups during the period of most rapid HIV risk reduction (1998–2005).

## Methods

### Data Source

The Manicaland HIV/STD Prevention Study (Manicaland Study) is a prospective general population cohort survey tracking trends in the HIV epidemic in twelve sites spread across three districts in Manicaland, Zimbabwe’s eastern province. The twelve sites represent four of the main socio-economic strata in Zimbabwe – small towns (2 sites), agricultural estates (4), roadside settlements (2) and subsistence farming areas (4) – and are enumerated in each round of the survey in a phased manner (one at a time) over periods of 18 months to two years. In each round, the data collected include information on socio-demographic characteristics and sexual behaviour. Dried blood spot specimens are collected and tested for the presence of HIV infection. Eligibility criteria included males aged 17–54 and females aged 15–44. Only one member of each cohabiting marital couple was selected at random. Participants were required to have stayed four nights in the household for the past month and at the same time one year ago. Further details of the survey methods are available in previous publications. [10], [16].

We used data from the baseline survey and the second follow-up survey of the Manicaland Study, which were collected between July 1998 and February 2000 and between July 2003 and August 2005, respectively. These rounds were selected because they spanned the period of greatest reduction in sexual risk behaviour in Manicaland [10] and in Zimbabwe in general [11], [12] and, therefore, provided an opportunity to compare changes in HIV infection and associated risk behaviours over time between religious groupings.

In the Manicaland Study, each participant was asked to identify the church that they belonged to. Churches identified in this way were then allocated to major religious groupings based on a categorization developed from the literature and using qualitative data collected in in-depth interviews carried out with 5 key informants in Zimbabwe. The key informant interviews were relatively brief, lasting approximately 15–30 minutes each. The key informants were leaders from the Evangelical Fellowship of Zimbabwe (a Pentecostal inter-denominational organization); the Scripture Union (an international Christian organization); the Africa Leadership and Management Academy (a Christian based college); Zimbabwe Assemblies of God Africa (ZAOGA) (a large local Pentecostal church); and Faith Ministries (another local Pentecostal church). These key informants were selected to provide a cross-section of the influential Christian-based church organizations in Zimbabwe. We were unable to interview leaders from Traditional and Spiritual churches so key informants were selected who could provide informative insight into not only Christian religions but also on Traditional and Spiritual religions in Zimbabwe.

Based on the results from the literature review and the in-depth interviews, the churches reported by the survey participants were divided into five major religious groupings: “Traditionalists”, “Spiritualists”, “Christians”, “Other” and “None”. The principal teachings and practices of these religious groupings that are relevant to the current study are summarized in Table 1.

**Table 1:** Principal teachings and practices of major religious groupings in Manicaland, Zimbabwe.

Teaching or practice	Traditional	Spiritual	Christian
Weekly meetings	No	Yes	Yes
Bible-based teachings	No	Partial	Yes
Polygyny condoned	Yes	Some groups	No
Alcohol consumption	Yes	No	Partial
Form of medicine	Herbs/ancestralspirits	Faith healing	Western
Condom use	Indifferent	No	Varies

### Data Analysis

The distributions of survey participants by religious grouping were calculated and compared between the two analysis periods to investigate possible changes over time. Then, the socio-demographic and behavioural characteristics of members of the different religious groupings were compared and again examined for possible changes over time. The social characteristics examined were age, education, marital status, and early marriage (men aged <24 years and women aged <18 years as defined by the Zimbabwe Demographic Health Survey 2010–2011 [17]). The behaviours compared were those previously associated with HIV infection, including: drinking alcohol on a regular basis (≥10 times per week), number of lifetime sexual partners, number of partners in the last year, and condom use. [10] Data on condom use were only collected and analysed at follow-up. Pearson’s chi-squared tests were used to assess statistically significant differences between religious groups and over time.

Univariate logistic regression analysis was conducted to identify associations between religious groupings, possible socio-demographic confounding factors and HIV infection status. Then, two multivariate logistic regression models were developed: (i) to test for independent associations between religious grouping and HIV infection status, and (ii) to investigate whether the associations observed were mediated by behaviour variables. The variable for early marriage was not included in the multivariate models because co-linearity between the marital status and being young when married variables could have resulted in over-fitting of the models.

To investigate the contribution of people who converted from one church to another to changes in HIV prevalence in the religious groupings during the study period, the proportions of church members at follow-up who reported having joined their current church in the last 5 years were calculated for each major religious grouping, and HIV prevalence was compared for new and long-term members.

Previously, data from the Manicaland Study have shown that HIV risk differs between men and women and over time, [10], [16] therefore, we stratified all analyses by survey round and by sex. All analyses were performed using STATA, version 10 (Stata Corp, College Station, Texas, USA).

Ethical Approval for the study was obtained from the Research Council of Zimbabwe (no. 02,187) and from the St. Mary’s Local Research Ethics Committee, London (HIV/GUM EC no. 03.66 R&D 03/SB/004E).

## Results

Data were available on 4,418 and 6,609 men aged 17–54 years and on 5,424 and 9,893 women aged 15–44 years in the baseline survey (1998–2000) and the follow-up survey (2003–2005), respectively. The participation rates at baseline were 76% for men and 78% for women; at follow-up, the participation rates were 77% and 86%.

The degree of missing data was limited (<15%) for both males and females except for the number of lifetime sexual partners in the baseline survey, where up to 22% was missing.

### Distribution of the Population between Religions

**Figure 1:**
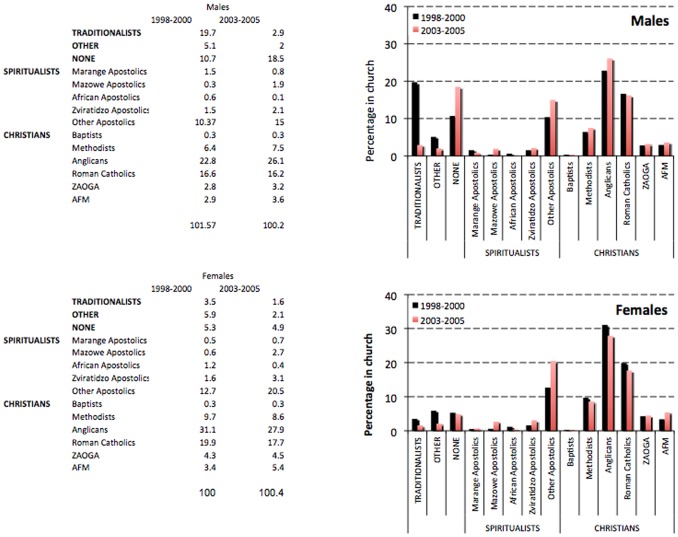
Ungrouped religious affiliations of survey participants.

Figure 1 shows the distribution of churches at each round of the survey before they were combined into the five main religious groupings used for the study. The Anglican and Roman Catholic churches had the most members amongst the various Christian churches whilst no single church stood out amongst the Spiritualist churches.

In the late 1990s, Christian churches were the most popular religious grouping for both men (54%) and women (70%) (Table 2 & Table3). Traditional religion was the second most common grouping amongst men (18%) followed by Spiritualist churches (13%) but, for women, Spiritualist churches were the second most common grouping (17%) and subscribers to Traditional religion were relatively few (3%). By the mid-2000s, membership of Christian churches had increased further in men (60%) but declined slightly amongst women (67%). However, Spiritualist churches had increased in popularity for both sexes (to 18% for men and 25%, for women). Only small numbers of participants in the follow-up survey reported subscribing to Traditional religion (3% of men; 2% of women) but there was an increase in the proportion of male respondents reporting no religious beliefs from 10% to 17%. However, none of the changes in religious groupings over time were statistically significant.

**Table 2:** Socio-demographic and sexual behaviour profiles of religious groups in 1998–2000 and in 2003–2005 in Manicaland, Zimbabwe: males.

		Traditional				Spiritual				Other				None				Christian			
		1998–2000		2003–2005		1998–2000		2003–2005		1998–2000		2003–2005		1998–2000		2003–2005		1998–2000		2003–2005	
Characteristic	Coding	%	n	%	n	%	n	%	n	%	n	%	n	%	n	%	n	%	n	%	n
Age-group	<25 years	33	253	19	35	49	291	45	483	55	113	44	52	48	209	33	345	55	1255	49	1696
	25–34 years	34	259	28	45	33	196	33	345	29	60	31	36	30	129	35	364	24	559	26	904
	≥35 years	33	257	53	85	18	106	22	234	16	34	25	29	22	97	32	341	21	489	25	869
Education	None/primary	51	403	43	70	35	211	26	303	21	44	23	29	37	163	37	392	26	602	19	764
	Secondary/higher	49	383	57	94	65	388	74	877	79	170	77	99	63	277	63	680	74	1742	81	3165
Marital status	Single	31	247	20	33	51	308	49	583	57	122	47	62	48	212	33	366	60	1415	56	2208
	Married	61	481	77	133	45	268	48	569	39	84	48	63	46	201	60	671	35	822	40	1592
	Divorced	6	46	2	4	3	20	2	27	3	6	4	5	4	19	6	62	4	87	3	115
	Widowed	2	12	1	2	1	3	1	17	1	2	1	1	2	8	1	15	1	31	1	41
Early marriage†	Later marriage	91	491	96	134	92	267	93*	570	91	84	91**	63	93	212	93	695	94	886	94	1649
	Early marriage	9	48	4	5	8	24	7	43	9	8	9	6	7	16	7	53	6	53	6	99
Drinks regularly	No	84*	660	81	140	95**	567	98**	1171	86**	183	98**	118	78	343	88*	979	79	1855	91	3608
	Yes	16	126	19	32	5	32	2	24	14	31	2	3	22	97	12	133	21	499	9	348
Lifetime partners	1	9	66	17	29	18**	90	29**	243	14**	24	32*	28	9	35	16	162	13	242	25	671
	2	10	73	16	27	13	63	20	167	9	15	13	12	12	44	18	183	13	235	17	456
	3	13	96	12	19	14	71	15	124	13	22	17	15	14	52	16	156	13	230	16	414
	4+	68	485	55	90	55	271	36	308	64	109	38	34	65	249	50	497	61	1122	42	1128
No. of sexual partners in last 12 months	0	15	115	10*	18	28*	172	39**	463	30*	62	39**	51	7	27	18	202	14	257	41	1610
	1	45	351	65	111	43	265	46	545	38	80	46	60	46	177	53	589	46	845	43	1703
	2+	39	306	25	43	28	168	15	187	31	164	15	20	47	180	29	323	40	741	16	642
Condom use with non-regular partner	Never			41	26			48	171			58	18			46	222			49	618
	Less than a year			13	8			12	43			7	2			7	35			10	132
	More than a year			46	29			40	140			35	11			47	224			41	518
Abstinence	Never had sex	6	47	5	8	17	96	29	340	17	37	31	41	12	50	10	112	21	480	32	1264
	Abstaining	28	210	20	34	34	194	24	284	34	72	18	24	31	130	25	282	33	757	25	969
	Not abstaining	66	505	75	128	49	284	47	555	45	96	51	66	57	240	62	696	46	1034	43	1679
Distribution of religions		18	786	3	172	13	599	18	1196	5	214	2	131	10	440	17	1114	54	2355	60	3956

** p<0.001.

* p<0.05.

Difference in behaviour variable in religious group versus Christians adjusted for age using logistic regression. Variables converted to binary variables:Variables converted to binary variables:

Lifetime sexual partners: 1&2 versus 3+; no. of sexual partners in 12 months: 0&1 versus 2+; condom use: never and less than a year vs. more than a year.

† Married before age 18 years.

**Figure 2:**
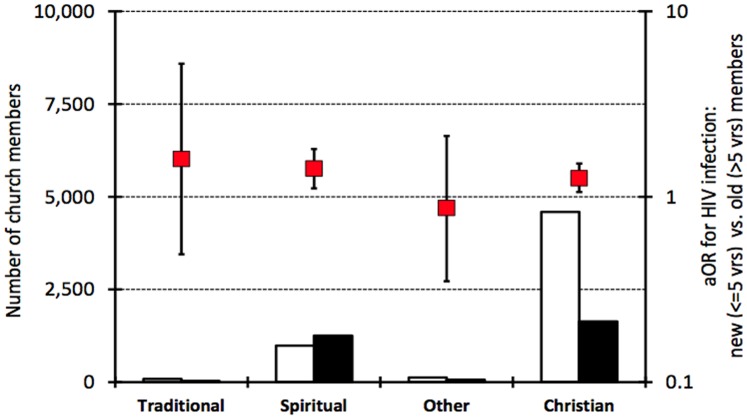
Differences in HIV prevalence between long-term and new female church members by major religious grouping (2003–2005). aOR, odds of HIV infection adjusted for age-group, education and marital status.

In the follow-up survey, amongst women, 26% of Christians, 56% of Spiritualists and 67% of members of other churches reported having joined their church in the last 5 years (i.e. since baseline) (Figure 2). Women who were converted to a Christian church were equally likely to have moved from another Christian church or a Spiritualist church (46% in each case), whilst those who were converted to a Spiritualist church were most likely to have moved from another Spiritualist church (54% vs. 36%). Marriage (36% for Christian churches and 22% for Spiritualist churches) and ‘better church beliefs’ (22% and 24%) were the most common reasons given for changing church. Sickness was cited more frequently as the main reason for changing church by women joining Spiritualist churches than by those joining Christian churches (10% vs. 2%).

Males in Spiritual churches were also more likely than those in Christian churches to have joined their church recently (49.4% vs. 21.0%). New members of both Christian and Spiritual churches were most likely to have joined from a Spiritual church (43% and 51%, respectively) and sizeable proportions had previously had no religion (24% and 16%).

### Comparison of the Socio-demographic and Behaviour Profiles of Members of Different Religions

For both sexes, subscribers to Traditional religion tended to be older than members of other religions, a difference that increased over time (Table 2 & Table 3). Marriage levels were high for both sexes across all religions.

**Table 3:** Socio-demographic and sexual behaviour profiles of religious groups in 1998–2000 and in 2003–2005 in Manicaland, Zimbabwe: females.

		Traditional				Spiritual				Other					None					Christian				
		1998–2000		2003–2005		1998–2000		2003–2005		1998–2000		2003–2005		1998–2000			2003–2005		1998–2000			2003–2005		
Characteristic	Coding	%	n	%	n	%	n	%	n	%	n	%	n	%		n	%	n	%		n	%	n	
Age-group	<25 years	41	70	32	39	47	371	44	943	46	142	51	92	37		96	48	184	44		1569	48	2593	
	25–34 years	34	59	35	44	31	249	35	754	31	95	28	51	40		105	32	122	27		978	29	1597	
	≥35 years	25	43	33	41	22	173	21	452	23	70	21	37	23		62	20	75	29		1016	23	1234	
Education	None/primary	76	136	57	64	58	490	46	1067	43	135	45	85	69		186	61	226	43		1609	35	2285	
	Secondary/higher	24	42	43	49	42	352	54	1239	57	181	55	103	31		82	39	144	57		2158	65	4157	
Marital status	Single	10	17	12	17	21	177	19	469	27	85	28	58	16		43	11	50	26		994	26	1692	
	Married	64	114	66	94	60	501	61	1479	53	168	57	117	56		149	62	266	55		2065	54	3596	
	Divorced	18	32	12	17	13	109	11	263	12	37	7	14	20		53	18	78	10		393	9	620	
	Widowed	8	15	10	15	6	55	9	225	8	26	8	16	8		23	9	37	9		315	11	710	
Early marriage†	Later marriage	94	152	100	125	98*	649	99	1940	98**	226	99**	146	97		219	97	370	97		2698	99	4876	
	Early marriage	6	9	0	0	2	16	1	27	2	5	1	1	3		6	3	11	3		73	1	48	
Drinks regularly	No	97	171	98	140	99	837	99**	2433	99*	297	100**	187	96		258	97	418	99		3731	99	6410	
	Yes	3	5	2	3	1.0	4	1	2	1	2	0	0	4		10	3	13	1		33	1	5	
Lifetime partners	1	60	96	61	79	64*	435	75**	1522	57**	139	72**	113	49**		117	54	215	66		1952	79	4075	
	2	17	27	25	32	19	126	17	335	25	62	16	26	18		44	21	84	19		561	14	732	
	3	8	13	5	6	8	57	5	98	8	20	8	12	12		28	9	34	6		201	4	178	
	4+	15	24	9	12	9	62	3	69	9	23	4	7	21		51	16	65	9		254	3	162	
No. of sexual partners in last 12 months	0	14	23	25*	36	14	99	35*	860	19*	46	32**	66	14		35	23	101	18		546	40	2630	
	1	79	126	71	102	81	553	63	1535	77	189	65	132	69		168	66	286	77		2303	59	3890	
	2+	7	11	4	5	5	33	2	39	4	10	3	6	17		40	10	44	5		147	1	93	
Condom use withnon-regular partner	Never			42*	5			67	127			79	15				58	52				67	281	
	Less than a year			0	0			7	13			5	1				5	4				6	24	
	More than a year			58	7			26	50			16	3				37	33				27	115	
Abstinence	Never had sex	8	13	9	13	17	150	17	391	21	62	23	47	9		23	7	28	20		724	22	1434	
	Abstaining	26	43	33	46	26	228	29	699	27	80	26	52	28		75	31	130	31		1152	31	2028	
	Not abstaining	61	102	58	81	55	472	54	1287	48	144	51	102	60		161	62	264	49		1771	47	3009	
Distribution of religions		3	178	2	143	17	842	25	2436	5	316	2	205	5		268	4	431	70		3767	67	6418	

** p<0.001.

* p<0.05.

Difference in behaviour variable in religious group versus Christians adjusted for age using logistic regression. Variables converted to binary variables:Variables converted to binary variables:

Lifetime sexual partners: 1&2 versus 3+; no. of sexual partners in 12 months: 0&1 versus 2+; condom use: never and less than a year vs. more than a year.

† Married before age 18 years.

For men, in the late 1990s, alcohol consumption was most common amongst those in Christian churches (21%) and with no religion (22%) and was least common amongst those in Spiritual churches (5%). By the mid-2000s, alcohol consumption had fallen amongst men in Christian churches (9%, p = 0.03) and was highest in men who followed the Traditional religion (19%, p = 0.6) (Table 2). For women, alcohol consumption was generally low with only a few of those subscribing to Traditional religion (3% at baseline) or with no religion (4%) reporting that they drank alcohol (Table 3).

Men following Traditional religion and men with no religion at baseline reported more sexual partners in their lifetime than those in Christian churches whilst men from Spiritual churches reported fewer partners than Christian men (Table 2). The men from all religions interviewed at follow-up reported smaller numbers of lifetime partners and fewer partners in the last 12 months than those interviewed at baseline. Men with no religion and those subscribing to Traditional religion continued to report more lifetime partners and reported more partners in the last 12 months than men from Christian churches. Men from Spiritual churches still reported fewer sexual partners over their lifetimes than those from Christian churches; however, Christian men now reported similar numbers of partners in the last 12 months to their Spiritualist counterparts.

As for men, women subscribing to Traditional religion and women with no religion reported higher numbers of partners than Christian women at baseline (Table 3). However, women from Spiritual churches reported similar numbers of partners to those from Christian churches. Again, lower numbers of sexual partners were reported in all religious groupings at follow-up. Women following Traditional religion and those with no religion continued to report higher numbers of past and recent partners than Christian women, whilst reported partner numbers in Christian and Spiritual churches remained similar.

For both men and women, those from Christian and Spiritual churches who reported non-regular sexual partners were equally likely to report consistent condom use (Table 2 & Table 3). Those following Traditional religion or with no religion reported somewhat higher condom use but the differences were not statistically significant.

### Comparison of HIV Prevalence between Religious Groupings in the Late 1990s

At baseline, in the univariate analysis (Table 4), men subscribing to Traditional religion (26.7% *vs.* 17.5%, p<0.001) or with no religion (23.8% *vs.* 17.5%, p<0.05) were more likely to be infected with HIV than those in Christian churches, whilst HIV prevalence in men in Spiritual churches was borderline significantly lower (14.4% *vs.* 17.4%, p = 0.076). After controlling for socio-demographic confounding factors, the differences between Traditional and no religion compared to Christian religion were reduced and no longer statistically significant. However, the lower HIV prevalence associated with membership of a Spiritual church became more pronounced and statistically significant (aOR = 0.7; 95% CI 0.50–0.86). After further adjustment for differences in alcohol consumption and number of lifetime sexual partners, the protective effect of membership of a Spiritual church was reduced and ceased to be statistically significant (aOR = 0.8*;* 0.60–1.06) – suggesting that the lower levels of sexual risk behaviour in these churches had contributed to their lower HIV prevalence.

**Table 4:** Comparison of HIV prevalence between religions over time, Manicaland, Zimbabwe: univariate and nested multivariate regression models for 1998–2000 and 2003–2005: males.

		Univariate							Model 1 (D+R)				Model 2 (D+R+B)			
		1998–2000			2003–2005			1998–2000			2003–2005		1998–2000		2003–2005	
Characteristic	Coding	OR	p-value	HIV+ (%)	OR	p-value	HIV+ (%)	aOR		p-value	aOR	p-value	aOR	p-value	aOR	p-value
Religion	Traditional	1.7	<0.001	26.7%	2.1	0.393	24.6%	1.2		0.160	1.3	0.183	1.2	0.122	1.6	0.142
	Spiritual	0.8	0.076	14.4%	1.0	0.094	12.7%	0.7		0.002	0.9	0.232	0.8	0.121	0.8	0.394
	Other	0.8	0.354	15.0%	1.0	0.979	13.0%	0.8		0.334	0.9	0.665	0.9	0.561	1.1	0.875
	None	1.5	0.002	23.8%	1.7	1.662	20.2%	1.3		0.077	1.2	0.031	1.3	0.111	1.2	0.270
	Christian	1		17.5%	1		13.2%	1			1		1		1	
Age-group	<25 years	1		4.8%	1		2.9%	1			1		1		1	
	25–34 years	10.0	<0.001	33.8%	8.5	<0.001	20.3%	6.5		<0.001	4.4	<0.001	4.4	<0.001	3.9	<0.001
	≥35 years	9.8	<0.001	33.3%	16.8	<0.001	33.5%	5.4		<0.001	7.6	<0.001	3.2	<0.001	7.8	<0.001
Education	None/primary	1.5	0.409	23.8%	1.9	<0.001	21.4%	0.9		0.365	0.9	0.116	1.0	0.040	0.9	0.648
	Secondary/higher	1		17.0%	1		12.6%	1			1		1		1	
Marital status	Single	1		7.4%	1		3.1%	1			1		1		1	
	Married	5.5	<0.001	30.5%	9.9	<0.001	24.1%	1.9		<0.001	2.6	<0.001	1.7	<0.001	2.6	<0.001
	Divorced	8.7	<0.001	41.0%	18.4	<0.001	37.2%	3.1		<0.001	5.2	<0.001	2.4	<0.001	3.9	<0.001
	Widowed	17.9	<0.001	58.9%	76.3	<0.001	71.0%	5.8		<0.001	29.8	<0.001	5.1	<0.001	17.4	<0.001
Drinks regularly	No	1		16.9%	1		14.1%						1		1	
	Yes	2.1	<0.001	29.6%	1.6	<0.001	20.9%						1.3	0.005	0.9	0.466
No. of sexual partners in lifetime	1	1		4.6%	1		10.1%						1		1	
	2	3.0	<0.001	12.4%	1.6	0.001	14.9%						2.5	0.001	1.2	0.563
	3	4.6	<0.001	18.1%	2.1	<0.001	19.3%						3.2	<0.001	1.7	0.120
	4+	8.4	<0.001	28.8%	3.3	<0.001	27.2%						5.1	<0.001	2.4	0.006
Condom use with non-regular partner	Never						19.3%								1.2	0.393
	Less than a year						16.7%								0.9	0.696
	More than a year						12.7%								1	

For women, as for men, the univariate results showed higher HIV prevalence amongst those following Traditional religion (35.4% *vs.* 24.1%, p<0.001) and those with no religion (37.5% *vs.* 24.1%, p<0.001) than for those in Christian churches (Table 5). These differences were reduced after adjusting for socio-demographic confounding factors but remained borderline statistically significant for Traditional religion (aOR = 1.4; 0.95–1.92) and significant for no religion (aOR = 1.5; 1.10–1.94). However, after further adjustment for sexual behaviour, the differences between women with no religion and those in Christian churches were reduced and ceased to be statistically significant (p = 0.4). Women in Spiritual churches had a similar HIV prevalence (25.6%) to women in Christian churches (24.1%), a pattern that was not affected by adjustment for differences in socio-demographic or behavioural characteristics (Table 5).

**Table 5:** Comparison of HIV prevalence between religions over time, Manicaland, Zimbabwe: univariate and nested multivariate regression models for 1998–2000 and 2003–2005: females.

			Univariate						Model 1 (D+R)				Model 2 (D+R+B)			
			1998–2000			2003–2005			1998–2000		2003–2005		1998–2000		2003–2005	
Characteristic	Coding		OR	p-value	HIV+ (%)	OR	p-value	HIV+ (%)	aOR	p-value	aOR	p-value	aOR	p-value	aOR	p-value
Religion	Traditional		1.7	0.001	35.4%	2.3	<0.001	32.6%	1.4	0.093	2.1	0.020	1.2	0.293	1.8	0.413
	Spiritual		1.1	0.357	25.6%	1.2	0.001	21.6%	1.0	0.998	1.2	0.014	1.0	0.962	1.5	0.057
	Other		1.2	0.166	27.6%	1.3	0.176	21.0%	1.1	0.352	1.5	0.065	1.1	0.377	1.1	0.920
	None		1.9	<0.001	37.5%	2.0	<0.001	30.7%	1.5	0.009	1.9	<0.001	1.1	0.414	1.1	0.675
	Christian		1		24.1%	1		18.3%	1		1		1		1	
Age-group	<25 years		1		15.8%	1		8.3%	1		1		1		1	
	25–34 years		3.6	<0.001	40.5%	5.2	<0.001	32.3%	2.2	<0.001	3.2	<0.001	1.6	<0.001	3.4	<0.001
	≥35 years		1.9	<0.001	26.4%	4.5	<0.001	28.4%	0.9	0.366	2.2	<0.001	0.7	0.005	2.2	0.007
Education	None/primary		1.4	0.004	29.2%	1.4	<0.001	23.1%	1.2	0.021	1.0	0.911	1.1	0.165	1.4	0.141
	Secondary/higher		1		22.2%	1		17.5%	1		1		1		1	
Marital status	Single		1		9.0%	1		4.9%	1		1		1		1	
	Married		3.1	<0.001	23.4%	4.3	<0.001	18.0%	2.3	<0.001	2.5	<0.001	1.1	0.553	1.3	0.310
	Divorced		10.1	<0.001	49.9%	12.0	<0.001	38.0%	7.3	<0.001	6.0	<0.001	2.0	<0.001	1.4	0.187
	Widowed		12.7	<0.001	22.7%	17.6	<0.001	47.2%	10.8	<0.001	14.1	<0.001	4.5	<0.001	4.3	<0.001
Drinks regularly	No		1		25.1%	1		19.9%					1		1	
	Yes		7.8	<0.001	72.2%	7.6	<0.001	65.2%					1.6	0.199	2.6	0.180
No. of sexual partners in ifetime	1		1		21.7%	1		18.3%					1		1	
	2		2.1	<0.001	36.6%	2.7	<0.001	37.9%					1.9	<0.001	2.2	<0.001
	3		3.6	<0.001	50.0%	3.6	<0.001	44.9%					3.0	<0.001	1.6	0.140
	4+		6.0	<0.001	62.5%	7.6	<0.001	63.1%					4.5	<0.001	5.0	<0.001
Condom use with non-regular partner	Never					0.8	0.537	42.3%							1.0	0.974
	Less than a year					0.8	0.139	36.3%							1.1	0.519
	More than a year					1.0		37.2%							1	

### Temporal Changes in Religion as a Determinant of HIV Infection

In the follow-up survey, HIV prevalence had fallen in all religious groupings for both sexes (Table 4 & Table 5). The drops in prevalence were greatest in Christians, such that, by the mid-2000s, levels of HIV infection in all other religious groups had increased relative to those in Christians.

In the univariate analysis, as in the late 1990s, men subscribing to Traditional religion (24.6%) and men with no religion (20.2%) had higher HIV prevalence than those in Christian churches (13.2%). However, the difference for Traditional religion ceased to be statistically significant after adjusting for differences in socio-demographic factors. For men in Spiritual churches, the lower HIV prevalence compared to men in Christian churches that had been seen at baseline was no longer present (Table 4).

Amongst women, in the mid-2000s, HIV prevalence remained highest in the Traditional religion (32.6% *vs.* 18.3% in Christian churches) and no religion (30.7%) groupings. As in the earlier period, these differences remained after accounting for differences in socio-demographic characteristics but ceased to be statistically significant after further adjustment for differences in sexual behaviour (Table 5). Unlike in the late 1990s, HIV prevalence in women in Spiritual churches was also higher than amongst women in Christian churches (21.6% *vs.* 18.3%, p = 0.001). The difference was reduced to borderline statistically significant after adjusting for differences in behaviour (aOR = 1.5; 95% CI 0.99–2.34).

### Comparison of HIV Prevalence between New and Long-term Church Members

Women who had joined a church in the last 5 years were more likely to be infected with HIV than long-term members after adjusting for differences in age, education, and marital status (20.4% vs. 19.9%, aOR = 1.32; 95% CI 1.15–1.51). This effect was seen in both Christian (18.5% vs. 17.8%, 1.26; 1.06–1.51) and Spiritualist churches (23.2% vs. 16.9%, 1.42; 1.11–1.81) and for all churches of origin (Figure 2).

For men, no differences were observed in HIV prevalence between new and long-term members, for any of the major religious groupings, after adjusting for differences in age, education and marital status (results not shown).

## Discussion

In eastern Zimbabwe, most men and women belong to orthodox Christian churches. This pattern continued during the early-mid 2000s, but membership of Spiritual churches increased and Traditional religion reduced in popularity (the latter, mainly due to population ageing).

In the late 1990s, we found that, for both men and women, Traditional religion and having no religious affiliation were associated with greater odds of being infected with HIV than belonging to a Christian church, whilst being a member of a Spiritualist church was protective for men and carried similar odds of HIV infection to Christian churches for women. The fall in HIV prevalence for both sexes in Manicaland over the subsequent five years [10] was observed in all religious groupings. However, the largest proportionate declines in HIV prevalence were recorded in Christian churches. As a consequence, membership of Christian churches became increasingly protective relative to other church groupings, with the initial advantage found amongst men in Spiritualist churches disappearing and women in these churches now suffering greater odds of HIV infection than women in Christian churches.

Most of the variation in HIV prevalence between religious groupings and over time was explained by differences in the socio-demographic characteristics of church members or by differences in levels and changes in sexual risk behaviour. In particular, the protective effect of membership of Spiritualist churches, found for men at the end of the 1990s, was accounted for by smaller numbers of lifetime sexual partners; whilst the reduction in this effect in the early-mid 2000s reflected greater declines within Christian churches in the rate of sexual partner acquisition over the subsequent five years.

These variations in sexual behaviour and in rates of reduction in risk behaviour, in turn, may be shaped by differences in church norms and teachings. For example, the smaller reduction in HIV risk behaviour found amongst Traditionalists could reflect health beliefs founded on Ancestral spirits and witchcraft – rather than Western explanations of sickness – and the central role of polygyny within Shona religion. [14] Polygyny is not approved of in Christian churches but, in Traditional religion and Spiritual churches, polygyny is widely accepted and sometimes encouraged. [18] Historically, polygyny was practical in that it ensured that a family had many children that could be used as labour to work on their land. [14] In recent times, levels of formal polygyny have been eroded by western Christian teachings, socio-economic development and other factors, although new forms have evolved such as the phenomena of ‘small houses’ in Zimbabwe. [19]

We have suggested previously that strictly enforced church rules prohibiting extra-marital sexual partnerships and alcohol consumption could provide protection against HIV infection within Spiritualist churches in Zimbabwe, even where polygyny continues to be practiced. [18] The main Spiritualist group in Manicaland that practices polygyny (the African Apostolic Church of Johane Marange) was not represented in the current study since church rules barred members from providing the dried blood spot samples required for HIV testing. Nevertheless, greater tolerance of polygyny together with underlying religious beliefs in the power of faith healing – rather than traditional or modern medicine – which are shared by most Spiritualist churches, may have restricted the reductions in numbers of sexual partners that occurred within these churches. In contrast, many Christian churches – particularly those with Missionary origins – are linked to provision and promotion of Western health beliefs and medicine including treatment of sexually transmitted infections. These churches are closely involved in national HIV control programmes and their teachings prohibiting or discouraging unfaithfulness and alcohol consumption – and reinforced through regular meetings – have been well attuned with national programme prevention messages. Therefore, it is quite plausible that members of Christian churches responded faster and more effectively in reducing their odds of HIV infection than those with no religion or in other major church groupings.

We observed extensive movements between churches. Overall, a larger fraction of Spiritualists than of Christians had joined their church recently (within the current study period). HIV prevalence was higher in new female converts than in long-term members – possibly, in part, due to ill-health as a reason for changing church – so these individuals could have contributed to the slower decline in HIV prevalence found in women in Spiritualist churches. However, unlike in the past, when Spiritual churches drew mainly from followers of Traditional religion [20], many of these new converts had joined from other Spiritualist churches so any such effect seems likely to be fairly small.

There have been surprisingly few previous detailed studies of associations between religious groupings and HIV risk in sub-Saharan Africa. In a study in Ghana, Takyi found that knowledge about HIV varied by religion but that there were no differences in sexual behaviour including condom use. [21] Similarly, in Malawi, Trinitapoli and colleagues found no differences in abstinence, faithfulness or condom use between members of Traditional, Christian, Muslim and non-religious groups, after adjusting for differences in gender, age and education. [7] In Zimbabwe, a study examining the influence of religion on attitudes, behaviours, and HIV infection among rural adolescent women between the period of 2007–2010 also found that the initial protective effect exhibited by Apostolics changed over time. This change was attributed to early marriage and the prohibition of members seeking medical testing and treatment. [22]

The strengths of this study include a large general population sample, representing four of the main socio-economic strata in Zimbabwe, and the availability of longitudinal data spanning a period of HIV decline associated with reductions in sexual risk behaviour. An important limitation of the serial cross-sectional analysis is that inferences about the direction of causality cannot be made since it is impossible distinguish whether a person’s religious affiliation preceded their HIV infection or behaviour. We examined differences between religious groupings in HIV prevalence and in changes in HIV prevalence over time. HIV prevalence is a useful indicator for assessing the relative burden of infection between time points and between different population groups. However, HIV prevalence is a measure of the cumulative rather than the recent risk of infection. Therefore, in assessing the contribution of differences in sexual behaviour to differences in HIV prevalence between groups and over time, we used a matching measure of cumulative behaviour (number of sexual partners in the lifetime). A comparison of changes in HIV incidence might have provided a clearer picture of the contributions of different religions to recent reductions in HIV risk. However, no data on HIV incidence were available in this study for the period prior to the reduction in HIV risk.

Social desirability bias and recall bias can distort self-reported data on sexual behaviour. In this study, we used a validated Informal Confidential Voting Interview method to reduce bias in reporting of sexual risk behaviours. [23] However, some residual bias may distort our comparisons of risk behaviour between religious groupings and over time. Importantly, despite these limitations, we did find that differences in HIV prevalence between religious groupings and over time could be explained by differences in sexual behaviour.

Participation rates were high overall, but the study suffered from selective exclusion of members of the African Apostolic Church of Johane Marange, who could have a different pattern of HIV risk to members of other major Spiritualist churches in eastern Zimbabwe. In a study in South Africa, Garner found that extra- and pre-marital sex was reduced in Pentecostal churches compared to other Christian churches due to high levels of indoctrination, religious experience, exclusion and socialisation. [8] Thus, HIV risk can vary amongst churches within the major religious groupings. In the current study, we found only small differences in HIV prevalence and associated behaviours between Roman Catholics and other Christians (results not shown). Nonetheless, more research is required to describe and investigate differences within religious groupings, to establish whether further changes in patterns of HIV risk between religious groups have occurred since the mid-2000s as well as to provide a deeper understanding of the different obstacles to behaviour change that exist between and within religions and insight as to how these obstacles might be addressed.

This study provides valuable information on the contribution of religion as a determinant of responses to the HIV epidemic in Zimbabwe. The data suggest that Christian churches, in particular, may have played an important role in facilitating the reductions in HIV risk that occurred in the country in the late 1990s and early 2000s. [11] The current study period pre-dates the introduction of antiretroviral therapy (ART) in Zimbabwe. However, policy-makers in Zimbabwe will need to take into account the different health beliefs in Spiritualist churches and Traditionalists when engaging with leaders of these religions to promote uptake of new treatment and prevention services such as ART and medical male circumcision as also suggested in a study conducted in Mozambique. [24] The results presented here suggest that strengthened engagement with the leaders of these religions could also be used to identify means of overcoming cultural obstacles to further reductions in risk behaviour.
